# A routine for the determination of the microstructure of stacking-faulted nickel cobalt aluminium hydroxide precursors for lithium nickel cobalt aluminium oxide battery materials

**DOI:** 10.1107/S1600576719016212

**Published:** 2020-02-01

**Authors:** Sebastian Bette, Bernd Hinrichsen, Daniela Pfister, Robert, E. Dinnebier

**Affiliations:** a Scientific Facility X-ray Diffraction, Max Planck Institute for Solid State Research, Heisenbergstrasse 1, Stuttgart, 70569, Germany; b Material Physics and Analytics Fachgebiet Anorganische Mikrostrukturen, BASF SE, RAA/OS – M300, Ludwigshafen, 67056, Germany; c Battery Materials Development, BASF SE, RCN/DL – M300, Ludwigshafen, 67056, Germany

**Keywords:** stacking faults, powder diffraction, *TOPAS*, battery materials, nickel cobalt aluminium (NCA) precursors

## Abstract

The microstructures of six stacking-faulted industrially produced cobalt- and aluminium-bearing nickel layered double hydroxide (LDH) samples that are used as precursors for Li(Ni_1−*x*−y_Co_*x*_Al_*y*_)O_2_ battery materials were investigated. Shifts from the brucite-type (AγB)□(AγB)□ stacking pattern to the CdCl_2_-type (AγB)□(CβA)□(BαC)□ and the CrOOH-type (BγA)□(AβC)□(CαB)□ stacking order, as well as random intercalation of water molecules and carbonate ions, were found to be the main features of the microstructures. A recursive routine for generating and averaging supercells of stacking-faulted layered substances implemented in the *TOPAS* software was used to calculate diffraction patterns of the LDH phases as a function of the degree of faulting and to refine them against the measured diffraction data. The microstructures of the precursor materials were described by a model containing three parameters: transition probabilities for generating CdCl_2_-type and CrOOH-type faults and a transition probability for the random intercalation of water/carbonate layers. Automated series of simulations and refinements were performed, in which the transition probabilities were modified incrementally and thus the microstructures optimized by a grid search. All samples were found to exhibit the same fraction of CdCl_2_-type and CrOOH-type stacking faults, which indicates that they have identical Ni, Co and Al contents. Different degrees of interstratification faulting were determined, which could be correlated to different heights of intercalation-water-related mass-loss steps in the thermal analyses.

## Abstract 2

Stacking faults and interstratification faults in a cobalt- and aluminium-bearing nickel layered double hydroxide used as a precursor for Li(Ni_1−*x*−*y*_Co_*x*_Al_*y*_)O_2_ battery materials were quantified by a combination of a grid-search approach and a recursive routine for generating and averaging supercells of stacking-faulted layered substances implemented in the *TOPAS* software.

## 1. Introduction   

**Figure 1:**
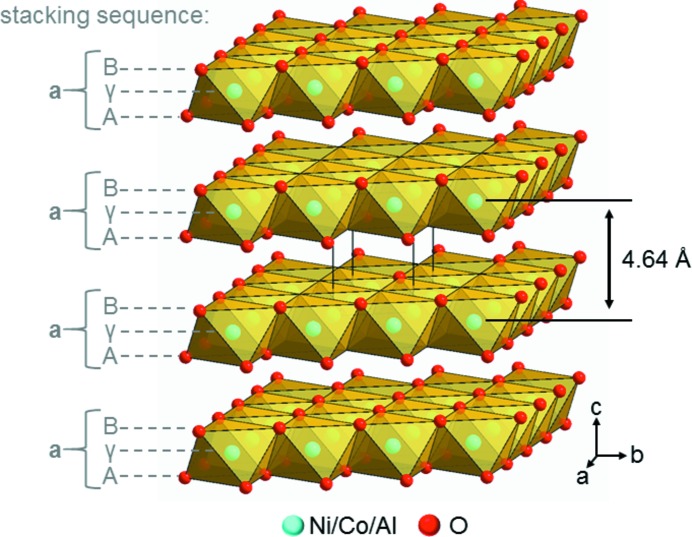
Packing diagram of the ideal brucite-type crystal structure of the NCA-precursor material, including the interlayer spacing. The positions of the cation layers are indicated by small Greek letters, the positions of the anion layers are indicated by capital Latin letters and the total layer positions are indicated by lower-case Latin letters.

Lithium-bearing transition metal oxides, Li*M*O_2_, such as LiCoO_2_ (Ohzuku & Ueda, 1994 ▸; Winter *et al.*, 1998 ▸), LiNiO_2_ (Dahn, 1991 ▸), Li(Ni_1−*x*_Co_*x*_)O_2_ (Delmas *et al.*, 1999 ▸) and Li(Ni_1−*x*−*y*_Co_*x*_Al_y_)O_2_ (NCA) (Castro-García, 2003 ▸; Chen *et al.*, 2004 ▸), are important constituents in state-of-the-art lithium-ion batteries. The NCA materials are particularly favourable owing to their high specific capacity, which enables the production of high-energy Li-ion cells (Rozier & Tarascon, 2015 ▸; Radin *et al.*, 2017 ▸; Kim *et al.*, 2018 ▸). Li(Ni_1−*x*−*y*_Co_*x*_Al_*y*_)O_2_ can be obtained by the calcination of a mixture of a lithium salt like LiOH·H_2_O and a cobalt- and aluminium-bearing nickel hydroxide as the NCA-precursor material (Cheralathan *et al.*, 2010 ▸), which is usually synthesized by co-precipitation (Kim & Kim, 2012 ▸; Kim *et al.*, 2018 ▸). The NCA-precursor material is a layered double hydroxide (LDH) phase that is structurally closely related to the brucite-type β-Ni(OH)_2_ (Kazimirov *et al.*, 2010 ▸). In the brucite-type structure, edge-sharing *M*O_6/3_ octahedra form layers perpendicular to the crystallographic *c* axis. The layers are stacked in an eclipsed fashion, leading to an …(AγB)□(AγB)□… stacking order, where the positions of the anion layers are indicated by capital Latin letters, the positions of the cation layers are indicated by small Greek letters and tetrahedral interlayer vacancies are indicated by a square (Fig. 1 ▸).

The brucite-type structure model of the NCA-precursor material, however, is strongly simplified and idealized. Owing to the partial substitution of divalent nickel by trivalent aluminium, either hydroxide must be partially replaced by oxide or additional anions have to be incorporated into the structure in order to maintain the charge balance (Zhao *et al.*, 2008 ▸; Luo & Dahn, 2009 ▸). The incorporation of additional anions like acetate (Poul *et al.*, 2000 ▸), nitrate (Taibi *et al.*, 2002 ▸) or halogenides (Taibi *et al.*, 2014 ▸) into the brucite-type structure of Ni(OH)_2_ is known to occur as an intercalation that leads to an increase of the interlayer distance of more than 2.5 Å. In addition to anions, water molecules can be intercalated between the brucite-type layers (Bode *et al.*, 1966 ▸; Braconnier *et al.*, 1984 ▸; Kamath *et al.*, 1997 ▸; Yang *et al.*, 2005 ▸), thus forming materials occurring as Ni(OH)_2−*x*_[A^*n*−^]_*x*/*n*_·*z*H_2_O which are commonly denoted as ‘α-Ni(OH)_2_’ (Hall *et al.*, 2014 ▸). Moreover the stacking order of the brucite-type layers can locally deviate from the …(AγB)□(AγB)□… pattern to an …(AγB)□(CβA)□(BαC)□… stacking order, *i.e.* the crystal structures of layered brucite-type materials often contain stacking faults (Delmas & Tessier, 1997 ▸).

The occurrence of stacking faults causes diffuse scattering that among other effects can lead to pronounced peak broadening (Welberry & Butler, 1994 ▸). This creates serious problems for the determination of the crystallite size and also impedes the structural characterization, as strongly broadened peaks almost merge with the background. In consequence, the correct extraction of peak intensities is challenging, or artificial higher lattice symmetries are found if strongly broadened peaks merge with the background and seemingly disappear. The complex diffraction line shapes of stacking-faulted samples can be modelled by anisotropic microstrain broadening models (Stephens, 1999 ▸; Leineweber, 2006 ▸) that are used to refine strongly idealized and averaged structure models of stacking-faulted substances (Todorova *et al.*, 2011 ▸), but information on the degree of faulting cannot be extracted from these approaches. The *DIFFaX* routine (Treacy *et al.*, 1991 ▸) enables the simulation of diffraction patterns as a function of the degree and type of faulting. By comparing the simulated with the measured diffraction patterns, some information on the degree and type of faulting, *i.e.* the microstructure, of stacking-faulted brucite-type Mg(OH)_2_ (Radha *et al.*, 2003 ▸) and Ni(OH)_2_ (Ramesh *et al.*, 2003 ▸; Ramesh & Kamath, 2008 ▸) can be extracted. This procedure, however, does not enable any refinement of a microstructural model against the measured data. The *FAULTS* routine (Casas-Cabanas *et al.*, 2016 ▸) connects recursive *DIFFaX* simulations with a Rietveld refinement (Rietveld, 1969 ▸) of the atomic parameters and thus enables further insights into the ideal faultless structure of stacking-faulted solids such as the layered Li(Ni_1−*x*−*y*_Co_*x*_Mn_*y*_)O_2_ material (Reynaud & Casas-Cabanas, 2017 ▸). The degree of faulting, however, cannot be directly refined from the measured data. Supercell approaches with a limited number of layers that are shifted freely (Wang *et al.*, 2011 ▸; Metz *et al.*, 2016 ▸) or constrained (Bette *et al.*, 2017 ▸; Mangelsen *et al.*, 2019 ▸) perpendicular to the stacking direction have been used to approximate the microstructures of stacking-faulted samples and allow a refinement of the degree of disorder to a certain extent. These approaches are time consuming (Bette *et al.*, 2015 ▸) and the refinement of complex faulting models against the measured data is challenging. Recently, a *DIFFaX*-like recursion routine (Coelho *et al.*, 2016 ▸) was implemented in the *TOPAS* software (Coelho, 2018 ▸). It was demonstrated that, by performing a series of simulations of diffraction patterns and each time using a different degree of faulting, the microstructural parameters of simple faulting scenarios can be refined against the measured data in a grid-search-like approach (Ainsworth *et al.*, 2016 ▸; Bette *et al.*, 2019 ▸).

In order to ensure the control of the electrochemical performance of the NCA battery materials which is directly related to the microstructure, detailed knowledge of the microstructures of NCA-precursor materials is required as they have a major impact on the lithium uptake during the calcination and they may template the microstructures of the calcinated materials. Furthermore the mass production of NCA-containing batteries creates a demand for a standardized routine quality control of the precursor material, which includes the quantification of the degree of faulting in the solids. In this study we present an efficient way to determine the complex microstructures of the NCA-precursor materials from measured laboratory X-ray powder diffraction (XRPD) data using the *DIFFaX*-like recursion routine implemented in the *TOPAS* software and a multidimensional grid-search approach.

## 2. Experimental   

### 2.1. Sample preparation   

In total, six different NCA-precursor samples, denoted as ‘L001’, ‘L002’, ‘L003’, ‘L004’, ‘L005’ and ‘L006’, were synthesized for the microstructural investigations. The syntheses were performed exactly according to methods described in the literature (Nakayama *et al.*, 2015 ▸; Ryoshi *et al.*, 2016 ▸), except that the Ni:Co:Al ratio was adjusted to 90:5:5.

The samples were synthesized by dissolving Ni(NO_3_)_2_·6H_2_O, Co(NO_3_)_2_·6H_2_O and Al(NO_3_)_2_·9H_2_O (all reagent grade, used without further purification) in a 90:5:5 stoichiometric ratio. Sodium hydroxide and ammonia solution were added for precipitation. During the precipitation, the mixture was heated at 333 K in a water bath and stirred with an overhead mixer. A calibrated pH meter was used to monitor the pH value of the suspension, which was kept between 10 and 11. After 6 h, the addition of the precipitation agent was completed and the mixture was allowed to cool to room temperature, while stirring was continued. After 12 h, the solid was filtered off and washed three times with hot deionized water. The precursor materials were dried in an oven at 423 K for 3 h.

### 2.2. Phase characterization   

#### 2.2.1. Elemental analyses   

Elemental analyses of carbon, hydrogen and nitrogen were performed with a Vario Micro Cube analyser (Elementar). For the sulfur analysis, the samples were combusted catalytically in a helium/oxygen atmosphere on a WO_3_/Al_2_O_3_ contact at approximately 1373 K. The sulfur from the sample was converted to a mixture of sulfur dioxide and sulfur trioxide. The sulfur trioxide was reduced in the mixture to sulfur dioxide on a copper catalyst at approximately 1123 K, and the sulfur dioxide was finally quantified with an IR detector for samples L001, L002 and L003 or a thermal conductivity detector for samples L004, L005 and L006.

#### 2.2.2. Vibrational spectroscopy   

The infrared spectra of the precursors were recorded in attenuated total reflection (ATR) geometry on a Fourier-transform infrared spectrometer from Thermo Nicolet Nexus equipped with a diamond ATR unit (GladiATR, Pike Technologies). The background spectrum was measured separately and subtracted.

#### 2.2.3. Scanning electron microscopy and energy dispersive X-ray spectroscopy   

Scanning electron microscopy (SEM) images were taken with a JEOL JSM-7500 TFE scanning electron microscope. Energy dispersive X-ray spectroscopy (EDX) was performed with an Oxford Aztech spectroscope using an acceleration voltage of 15 kV and a working distance of 9 mm.

#### 2.2.4. Thermal analyses   

Thermal analyses were carried out using an STA 449 F5-Jupiter (Netzsch) device for thermogravimetric (TG) measurements. In each case, the sample (∼20 mg) was placed in an Al_2_O_3_ crucible and heated from 303 to 773 K with a heating rate of 2 K min^−1^ in a 50 ml min^−1^ Ar stream. An initial hold for 30 min was performed at 303 K in order to remove adherent water.

#### 2.2.5. Laboratory X-ray powder diffraction   

XRPD patterns were collected at room temperature on a laboratory powder diffractometer in Debye–Scherrer geometry [Stadi P-Diffraktometer (Stoe), Mo *K*α_1_ radiation from primary Ge(111) Johann-type monochromator, Mythen 1K detector (Dectris)]. The samples were sealed in 0.5 mm-diameter borosilicate glass capillaries (Hilgenberg glass No. 0140), which were spun during the measurements. Each pattern was measured in a 2θ range from 2.0 to 70°, applying a step size of 0.01° and a total scan time of 10 h. The program *TOPAS 6.0* (Coelho, 2018 ▸) was used for all Rietveld-based (Rietveld, 1969 ▸) refinements of the microstructures of the precursor materials. Chebychev polynomials of sixth order were used to model the background, and the peak profile was described by applying the fundamental parameter approach as implemented in *TOPAS* (Cheary *et al.*, 2004 ▸). The procedure for optimizing the microstructure of the samples is described in detail in Section 3.

#### 2.2.6. Computation   

All operations using the *TOPAS* software were performed on a 64-bit Windows 7 desktop PC with a dual core 2.6 GHz (Intel Xeon CPU E5-2660 v3) processor setup and 32.0 GB RAM.

## 3. Results and discussion   

### 3.1. Phase characterization   

**Figure 2:**
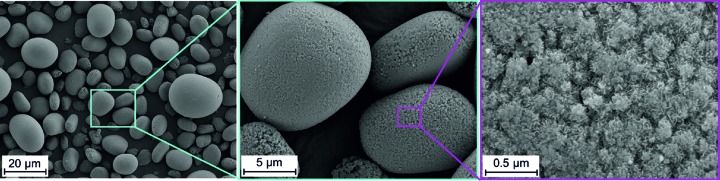
SEM images of the NCA-precursor material sample L001 as a representative for all samples.

**Figure 3:**
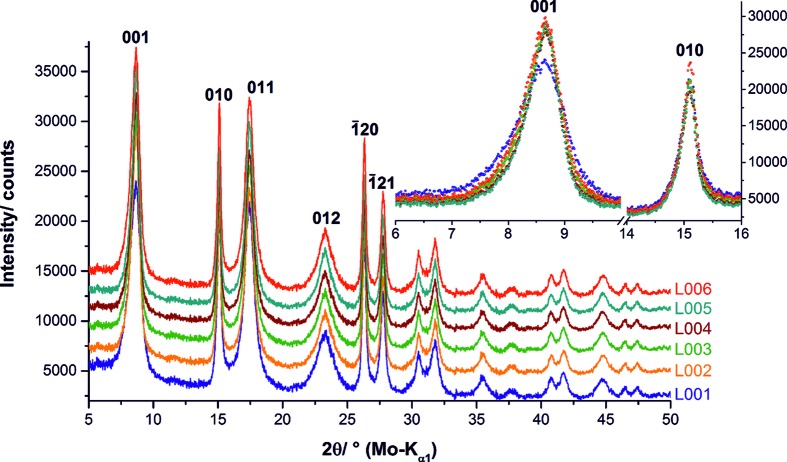
Measured XRPD patterns of the NCA-precursor materials. The patterns in the main part are stacked by adding 2000 counts to each one; the patterns in the inset are not stacked.

The synthesized NCA-precursor materials form spherical aggregates of very small nanometre-sized crystallites (Fig. 2 ▸). The chemical analyses revealed almost identical Ni, Co and Al contents for all samples (Table 1 ▸). In order to account for carbonization, the total carbon content was also analysed and found to be <0.15 wt% for all samples. In the measured diffraction pattern (Fig. 3 ▸) all peaks can be assigned to the brucite-type NCA-precursor material. The reflection positions and most peak shapes are nearly identical for all materials, except for sample L001 (blue pattern). The 001 reflection is significantly broader in the diffraction pattern of L001 than in the patterns of the other samples. The 010 reflection, however, exhibits the same shape for all investigated samples. This points to microstructural differences between sample L001 on the one side and samples L002, L003, L004, L005 and L006 on the other.

### 3.2. Derivation of microstructural features in the NCA-precursor materials   

**Figure 4:**
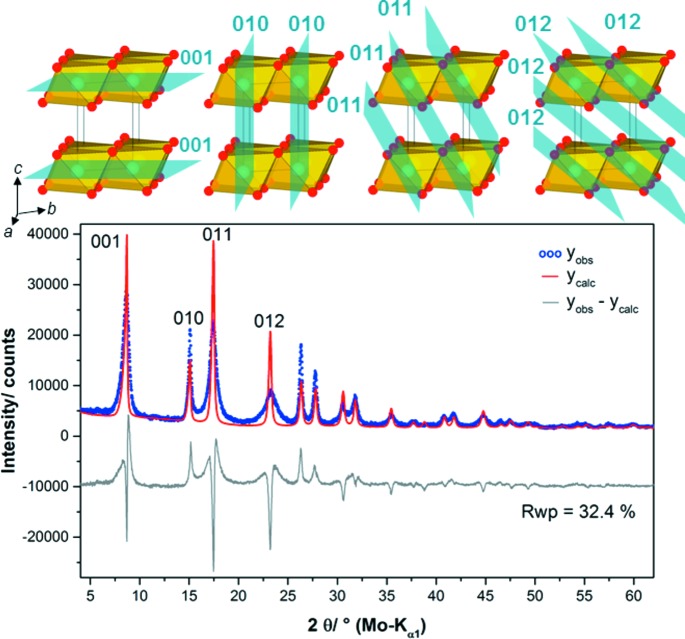
Graphical result of a Rietveld refinement of the diffraction pattern of the NCA-precursor material sample L002 using a faultless brucite-type structure model. The 010 reflection was used to model the contribution of the crystallite size to the peak shapes. Selected reflection indices and the positions of the related lattice planes in the crystal structure (top) are given.

The reflections in the diffraction patterns of the NCA-precursor materials exhibit a different degree of peak broadening: the 010 reflection is sharp, the 001 and 011 reflections are slightly broadened, and the 012 reflection is strongly broadened. Therefore, a Rietveld refinement (Rietveld, 1969 ▸) of the diffraction pattern of sample L002 led to a clear misfit (Fig. 4 ▸, bottom). While the peak shape of the 010 reflection can be modelled properly, the misfit of the 001, the 011 and in particular the 012 reflection is significant. The 010 reflection is related to lattice planes perpendicular to the layers and therefore this reflection is mainly affected by the layer constitution. In contrast, the 011 reflection is also governed by the relative layer orientation and the 001 reflection by the interlayer distance (Fig. 4 ▸, top). As the peak broadening can be attributed to the loss of coherence in the crystal structure (Welberry & Butler, 1994 ▸), it can be concluded that defects are apparent in the precursor materials that effect both the relative layer orientation and the interlayer distance. The absence of characteristic trigonal-shaped Warren peaks (Warren, 1941 ▸) also suggests that the faulting does not occur in a fully random fashion.

**Figure 5:**
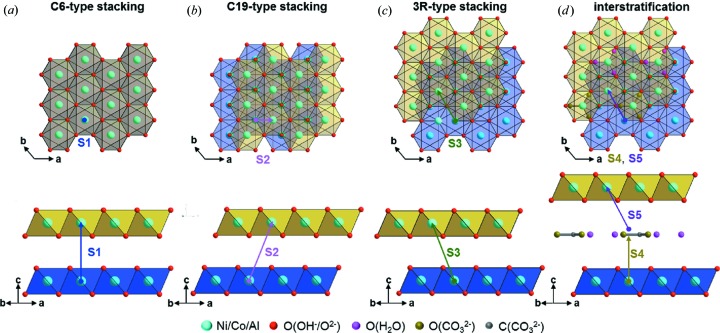
Stacking types that are considered as being apparent in the microstructure of the NCA-precursor materials; the bottom layers are indicated with yellow and the top layers with blue octahedra. (*a*) Brucite-type C6 stacking, (*b*) CdCl_2_-type C19 stacking, (*c*) CrOOH-type 3R stacking and (*d*) interstratification of water/carbonate. The coordinate systems refer to the transformed unit cells with *a* = *b* = 5.246 Å, *c* = 4.647 Å, α = β = 90° and γ = 120° (Fig. S1). The possible layer-to-layer transitions and the related stacking vectors are presented in Fig. 6.

In a brucite-type (C6-type of CdI_2_-type) structure the layers are stacked in an (AγB)□(AγB)□(AγB)□ fashion, with the protons of the hydroxide ions located in tetrahedral interlayer voids (Fig. 5 ▸). An (AγB)□(CβA)□(BαC)□ stacking order, *i.e.* C19-type stacking (CdCl_2_ type), also provides tetrahedral interlayer voids (Fig. 5 ▸) and therefore an equivalent packing of the anion and cation substructures. Both the partial substitution of nickel cations with smaller aluminium ions (Shannon, 1976 ▸) and synthesis conditions that are far from thermodynamic control can lead to C19-type stacking faults in the C6-type stacking order. Faultless crystalline β-Ni(OH)_2_ can be obtained by precipitation from very diluted salt solutions, and the synthesis procedure includes several ageing and washing steps (Palmer & Gamsjäger, 2010 ▸). As the applied synthesis conditions (see Section 2.1) strongly deviate from this procedure, the occurrence of this type of fault for thermodynamic reasons appears to be likely. In addition, the partial replacement of divalent nickel by trivalent aluminium or trivalent cobalt (the partial oxidation of Co^2+^ during the co-precipitation cannot be excluded) leads to an excess of positive charge in the cation substructure. This can be compensated by a partial deprotonation of hydroxide, yielding oxide. The oxide ions are possible acceptors of strong interlayer O—H⋯O hydrogen bonds. These bonds can only be formed when hydroxide and oxide ions of adjacent layers are located in direct opposition to each other. This is realized by an (AγB)□(BαC)□(CβA)□ stacking order, which can be denoted as 3R-type or CrOOH-type stacking (Fig. 5 ▸). C6-type faults in a 3R-type stacking were indeed observed in CoOOH (Kudielka *et al.*, 2017 ▸) and in NiCl(OH) (Bette *et al.*, 2015 ▸). Charge balance can also be maintained by the incorporation of anions, like carbonate, into the interlayer space, which leads to an increase of the interlayer distance (Fig. 5 ▸). This was observed in the crystal structure of takovite [Ni_1.869_Al_1.131_(OH)_6_(CO_3_)_1.020_; Mills *et al.*, 2013 ▸]. Neutral water molecules can also be intercalated between the brucite-type layers as in the crystal structures of iowaite {[Mg_4_Fe(OH)_10_Cl(H_2_O)_3_]_0.6_; Allmann & Donnay, 1969 ▸} and hydrotalcite {[Mg_4_Al_2_(OH)_12_CO_3_(H_2_O)_3_]_0.5_; Allmann & Jepsen, 1969 ▸}.

**Figure 6:**
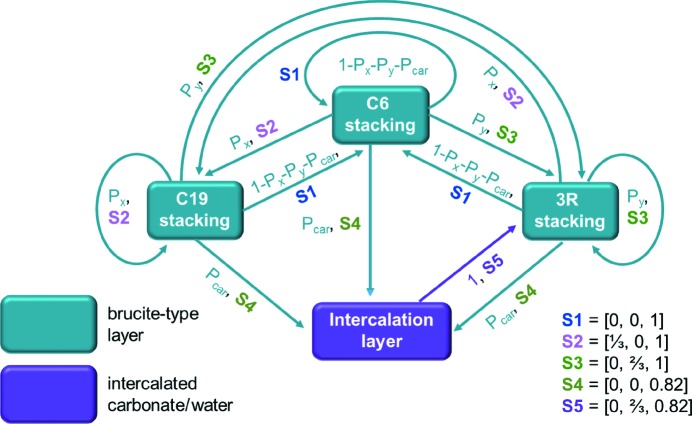
Scheme of the layer types and possible layer-to-layer transitions in the microstructure of the NCA-precursor materials. The stacking vectors refer to the transformed unit cell with *a* = *b* = 5.246 Å, *c* = 4.647 Å, α = β = 90° and γ = 120° (Fig. S1).

A microstructural model was created that includes C19-, C6- and 3R-type stacking and the intercalation of carbonate or water. For modelling of the carbonate intercalation layer the crystal structure of takovite was used. In order to ensure a minimum distance between interlayer carbonates, the brucite-type unit cell of the NCA-precursor material was transformed into a larger triclinic cell with trigonal metric (supporting information, Fig. S1). A schematic illustration of the microstructural model is presented in Fig. 6 ▸ and the corresponding transition probability matrix is shown in Table S1 (top) in the supporting information. The occurrence of C19-type stacking within the C6-type stacking order of the precursor material is described by the transition probability *P_x_*, the occurrence of 3R-type stacking by the transition probability *P_y_* and the occurrence of an intercalation layer by the transition probability *P*_car_. In the microstructural model all transitions between the different stacking patterns and within a certain stacking order are allowed, except for the intercalation layer. As an intercalation of two layers of water or carbonate is unlikely and potentially leads to exfoliation, each intercalation layer must be followed by a brucite-type layer stacked in the 3R-type fashion. The carbonate ion was oriented analogously to the orientation apparent in the structures of takovite and hydrotalcite in a way that provides short distances between the carbonate-related oxygen sites and hydroxide-related oxygen sites of the adjacent brucite-type layers (supporting information, Fig. S1).

### 3.3. Development of a method for global optimization of the microstructure   

An optimization of the microstructural parameters, *i.e.* the transition probabilities *P_x_*, *P_y_* and *P*_car_, was carried out for all samples. The applied routine is demonstrated in detail for sample L002. The microstructures of the samples were approximated using a supercell approach. Recently, a routine was published for the *TOPAS* software that enables the creation and averaging of a large number of supercells, each containing many layers (Coelho *et al.*, 2016 ▸). This results in a pseudo-recursive approach that is comparable to the *DIFFaX* routine (Treacy *et al.*, 1991 ▸). As the transition probabilities cannot be refined directly, the routine has to be carried out multiple times using a different set of transition probabilities, each time while monitoring the *R*_wp_ value. This results in a grid-search-like optimization of these parameters. An automated variation of the transition probabilities can be successfully achieved by an external Python script that executes the *TOPAS* input file multiple time and modifies the transition probabilities (Ainsworth *et al.*, 2016 ▸). In the supporting information (S2), multi-dimensional grid searches are demonstrated by exclusively using the *TOPAS* macro language.

#### 3.3.1. The obtained parameter spaces and refinements   

**Figure 7:**
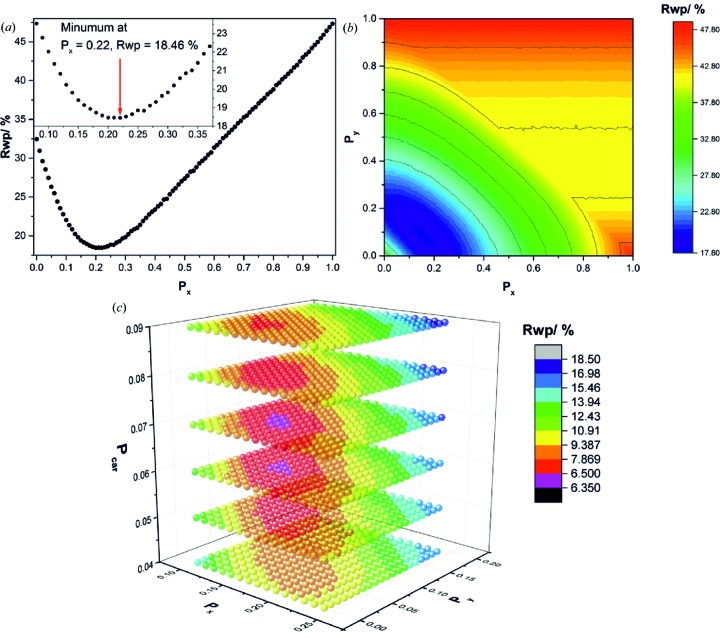
Results of the optimization of the transition probabilities describing the microstructure of the NCA-precursor sample L002 by grid-search algorithms. (*a*) One-dimensional parameter space including the probability of C19-type faults, (*b*) two-dimensional parameter space including the probabilities of C19- and 3R-type faults, and (*c*) three-dimensional parameter space including the probabilities of C19-, 3R- and interstratification-type faults.

**Figure 8:**
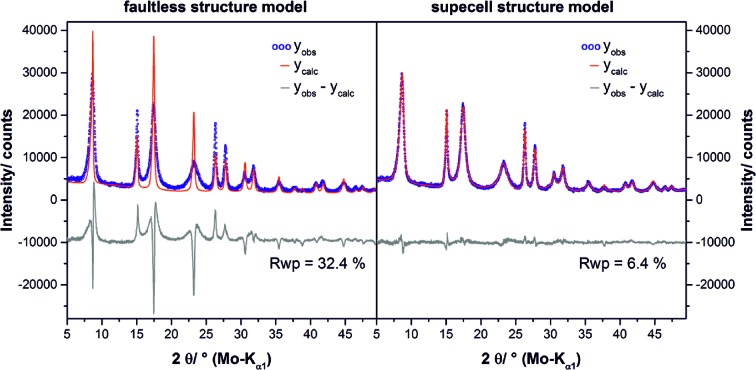
Comparison between the graphical results of the final Rietveld refinement of the NCA-precursor sample L002 by using the faultless brucite-type structure and by averaging 100 supercells with 500 layers each and using transition probabilities given in Table 2.

In Fig. 7 ▸ the graphical results of the (*a*) one-, (*b*) two- and (*c*) three-dimensional grid-search optimizations of sample L002 are presented. A comparison of the final fit using the faultless structure model and the supercell model that was obtained by the three-dimensional grid search is presented in Fig. 8 ▸. The evolution of the fits depending on the dimension of the applied grid is shown in Fig. S2. In the one-dimensional grid search, C19-type faults in the C6-type stacking of the NCA-precursor material were incorporated by using the transition probability *P_x_*. An initial increase of *P_x_* leads to a steep decrease of the *R*_wp_ value until at *P_x_* = 0.22 a global minimum is achieved [Fig. 7 ▸(*a*)]. Further increasing *P_x_* yields a higher *R*_wp_ value, which is at *P_x_* ≃ 1, *i.e.* almost pure C19-type stacking, significantly higher than for the faultless brucite-type structure. The final Rietveld refinement using a C19-type fault probability of *P_x_* = 0.22 [Fig. S2(*b*), straight red and grey lines] leads to a significantly better modelling of the peak shapes of the 011 and 012 reflections than using the faultless structure (dotted green and violet lines). There is, however, still a large misfit of the 001 reflection, and the shapes of the 011 and 012 reflections are not adequately described. By including 3R-type faults in the microstructure model, the global minimum of the parameter space is shifted towards lower *P_x_* values. Around *P_x_* = 0.18 and *P_y_* = 0.06, an ellipsoid-shaped minimum is present in the two-dimensional parameter space [Fig. 7 ▸(*b*)]. The extension of the parameter space only leads to a small improvement of the *R*_wp_ value from 18.4 to 17.9%, with the 011 reflection being slightly better but not perfectly modelled [Fig. S2(*c*)]. In the three-dimensional parameter space that also includes random intercalation of carbonate/water described by the parameter *P*_car_, a rather sharp global minimum is located at *P_x_* = 0.15, *P_y_* = 0.06 and *P*_car_ = 0.07 [Fig. 7 ▸(*c*)]. The inclusion of interstratification-type faults leads to a good fit of the powder pattern [Fig. S2(*d*)] with acceptable residual criterion (*R*_wp_ = 6.4%). In particular, the shape of the 001 reflection is modelled properly and the fit of the 011 and 012 reflections is also significantly improved.

### 3.4. A critical evaluation of the method   

**Figure 9:**
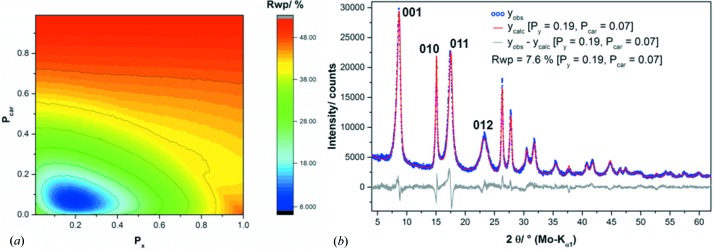
Results of the optimization of the transition probabilities describing the microstructure of the NCA-precursor sample L002 by grid-search algorithms. (*a*) Two-dimensional parameter space including the probabilities of C19- and interstratification-type faults with (*b*) the graphical result of the Rietveld refinement at the global minimum.

Despite the fact that the three-dimensional grid-search optimization led to a sharp minimum in the *P_x_*–*P_y_*–*P*_car_ parameter space and to a good fit of the measured powder pattern, the approach must be evaluated critically. First, the necessity to incorporate 3R faults into the microstructure is evaluated, as the extension from a one- to a two-dimensional parameter space only yielded a slight improvement of the fit (Fig. 8 ▸). Hence a two-dimensional grid search was performed in the *P_x_*–*P*_car_ parameter space, which led to a sharp minimum at *P_x_* = 0.19 and *P*_car_ = 0.07 [Fig. 9 ▸(*a*)]. The final Rietveld refinement at the global minimum yields acceptable residual criterion (*R*_wp_ = 7.6%) and a good graphical result for the fit [Fig. 9 ▸(*b*)]. There is, however, a considerable misfit of the 011 reflection. A comparison with the graphical result of the final Rietveld refinement using the global minimum of the three-dimensional parameter space reveals that the incorporation of 3R-type faults into the microstructure model leads to a significant improvement of the fit of the 011 reflection and is therefore necessary for a complete description of the microstructure of the NCA precursors.

**Figure 10:**
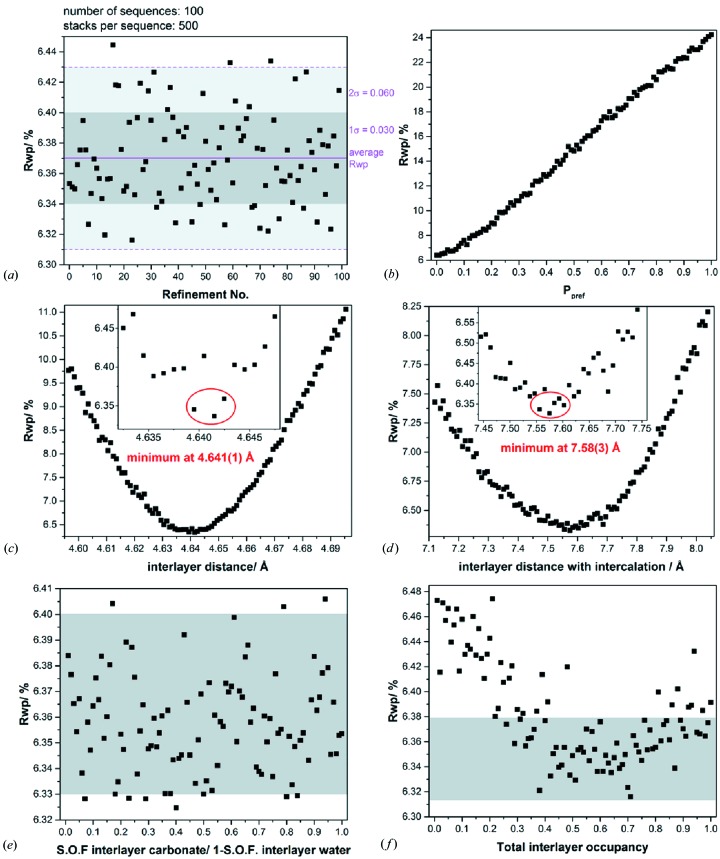
(*a*) Random distribution of the *R*_wp_ factors for 100 recursively created and averaged superstructures of the NCA-precursor material. (*b*) Evolution of the *R*_wp_ value after introducing into the microstructure model and increasing *P*_pref_, which describes the probability of an intercalation layer occurring after a fault in the stacking of the brucite-type layers. Grid-search optimization of the interlayer distance (*c*) between brucite-type layers and (*d*) between a brucite-type and an intercalation layer. Grid-search optimization (*e*) of the split occupancy of water and carbonate and (*f*) of the total occupancy of the intercalation layer. The grey background in (*e*) and (*f*) indicates the random variation of the *R*_wp_ value.

As the creation of stacking sequences is based on a random number generator and as only a limited number of stacking sequences were averaged, the fitting results can vary slightly for a given set of transition probabilities. Fig. 10 ▸(*a*) displays the *R*_wp_ values that were obtained after the measured diffraction pattern was fitted 100 times using averaged supercell models that were created with the same set of transition probabilities. The resulting *R*_wp_ values show a maximum variation of ±0.06% (2σ). Hence, all sets of transition probabilities that are located in the range of *R*_wp_ = (6.32–6.44)% can represent the global minimum of the *P_x_*–*P_y_*–*P*_car_ parameter space (supporting information Fig. S3). In consequence, the optimized microstructural parameters should be given as a range *P_x_* = 0.15 (1), *P_y_* = 0.06 (1), *P*_car_ = 0.07 (1) rather than as discrete values. By increasing the number of layers, *i.e.* ‘the number of stacks per sequence’, or increasing the number of averaged stacking sequences, *i.e.* ‘the number of sequences’, the random variation of the *R*_wp_ value can be reduced (supporting information Fig. S4). An increase in the number of layers per sequence from 500 to 1500 reduces the random variation of the *R*_wp_ value from ±0.060% (2σ) to ±0.034% (2σ) [supporting information Figs. S4(*a*)–4(*c*)]. This, however, is achieved at the expense of an increase of the average iteration time per step by a factor of three from 4.8 to 15.1 s (supporting information Fig. S5). It is also not good practice to use a supercell that significantly exceeds the domain size. Increasing the number of averaged supercells from 100 to 300 leads to a higher reduction of the random variation of the *R*_wp_ value from 0.060% (2σ) to ±0.032% (2σ) [supporting information Figs. S4(*d*)–S4(*f*)] at the expense of a smaller twofold increase of the average iteration time per step from 4.8 to 9.8 s (supporting information Fig. S5). Note that the three-dimensional grid search in the space of the microstructure parameters always leads to the same global minimum in a range of *P_x_* = 0.15 (1), *P_y_* = 0.06 (1), *P*_car_ = 0.07 (1), as long as at least 100 supercells containing at minimum 500 layers each are averaged.

The considered C19-type, 3R-type and intercalation faults represent all types of stacking faults that can be expected in the C6-type stacking of the NCA-precursor material. Nevertheless, the microstructure can be more complex. As each stacking fault represents a phase boundary with associated stacking-fault energy (Lecroisey & Pineau, 1972 ▸; Lee *et al.*, 2001 ▸; Hamada *et al.*, 2013 ▸; Rafaja *et al.*, 2014 ▸), there can be a preference for an intercalation after a 3R- or C19-type fault has occurred (supporting information Fig. S6). An additional microstructural parameter, *P*_pref_, was included in the model, which describes the preference for an intercalation after the occurrence of a stacking fault (Table S1, bottom). The parameter was implemented in such a way that the global probability of intercalation faults remains constant. At *P*_pref_ = 0, this parameter does not contribute to the microstructure of the sample and at *P*_pref_ = 1 an intercalation occurs preferentially after each stacking fault. A one-dimensional grid search in the parameter space of *P*_pref_ was performed at the global minimum (*P_x_* = 0.15, *P_y_* = 0.06 and *P*_car_ = 0.07) of the three-dimensinal parameter space that has been optimized so far. An increase of *P*_pref_ starting from 0 also leads to an increase of the *R*_wp_ value [Fig. 10 ▸(*b*)]. This increase becomes steeper when *P*_pref_ exceeds 0.1. Accordingly there is no preference for an intercalation after a stacking fault, as this leads to worse agreement factors.

By including the stacking faults and the random intercalation in the structural refinement, a few parameters, *e.g.* the interlayer distance and the intercalation-broadened interlayer distance, cannot be refined classically. Hence the interlayer distance was optimized by a one-dimensional grid search at the global minimum of the microstructure parameters (*P_x_* = 0.15, *P_y_* = 0.06 and *P*_car_ = 0.07). The graphical result is presented in Fig. 10 ▸(*c*). The grid shows a sharp global minimum at an interlayer distance of 4.641 Å. Owing to the statistical fluctuation of the results [Fig. 10 ▸(*a*)], the interlayer distance should be rather given as 4.641 (1) Å. In another one-dimensional grid search at the global minimum of the microstructure parameters (*P_x_* = 0.15, *P_y_* = 0.06 and *P*_car_ = 0.07), the interlayer distance after intercalation of water/carbonate was optimized. The grid [Fig. 10 ▸(*d*)] exhibits a slightly broadened minimum at 7.58 (3) Å. The grid search optimization of the microstructure parameters *P_x_*, *P_y_* and *P*_car_ (see Section S2.3) was performed again with the optimized interlayer distances, which led to the same global minimum.

In all grid searches that have been performed, the intercalated interlayer species was modelled by a carbonate ion with large displacement parameters for all atomic sites in order to account for positional disorder. As water molecules can be intercalated besides carbonate ions, an additional grid search was performed at the global minimum of the microstructure parameters (*P_x_* = 0.15, *P_y_* = 0.06 and *P*_car_ = 0.07) in order to investigate if interlayer carbonate can be distinguished from interlayer water. Layers of intercalated water were modelled analogously to the structure of iowaite {[Mg_4_Fe(OH)_10_Cl(H_2_O)_3_]_0.6_; Allmann & Donnay, 1969 ▸}, *i.e.* water-related oxygen sites were situated directly in opposition to the hydroxide ions of adjacent layers. The water layers were incorporated into the carbonate layers and the site occupancy factors were optimized in the one-dimensional grid search [Fig. 10 ▸(*e*)]. Throughout the whole grid only the random variation of the *R*_wp_ value [Fig. 10 ▸(*e*), grey shaded area] can be observed [Fig. 10 ▸(*a*)], *i.e.* by applying this routine, intercalated water cannot be distinguished from intercalated carbonate. For this purpose, complementary methods like IR spectroscopy or thermal analyses (see Section 3.6) have to be used.

In the case of an intercalation of water and/or carbonate, the intercalation layers do not necessarily have to be fully occupied, as partial occupation of intercalated molecules or ions was found in the structures of iowaite (Allmann & Donnay, 1969 ▸), takovite (Mills *et al.*, 2013 ▸) and hydrotalcite (Allmann & Jepsen, 1969 ▸). Hence the total occupancy of the intercalation layers was optimized in an additional one-dimensional grid search at the global minimum of the microstructure parameters (*P_x_* = 0.15, *P_y_* = 0.06 and *P*_car_ = 0.07). In this grid search, the total occupancy of the intercalation layer was incrementally increased from 0 to 1 [Fig. 10 ▸(*f*)]. An increase of the interlayer occupancy beginning from 0 initially leads to a slight improvement of the *R*_wp_ value. At an interlayer occupancy of ∼0.75 a very broad minimum is reached, and when the occupancy exceeds 0.85 the *R*_wp_ value slightly increases [Fig. 10 ▸(*f*)]. Note, however, that taking the random variation of the *R*_wp_ value into account [Fig. 10 ▸(*f*), grey shaded area] total interlayer occupancies in a range between 0.4 and 1.0 yield equivalent refinements. In consequence, it can be concluded that the interlayer space is occupied to more than 50% when an intercalation is occurring.

### 3.5. Application of the routine to other samples   

The microstructural parameters of all six NCA-precursor samples were optimized with the routine presented in Section 3.3. The graphical results of the refinements are presented in Fig. S7 in the supporting information. In Table 2 ▸ a comparison of the global minima of the three-dimensional parameter space is presented. All investigated NCA precursors exhibit almost the same percentage of C19 (14 ± 1%) and 3R faults (6–7%), which would be expected because of the similar Ni, Co and Al contents of the materials (Table 1 ▸). The percentage of interstratification faults is also almost identical for all precursor samples (6–7%), except for sample L001. Sample L001 exhibits significantly more interstratification faults (10 ± 1%) than the other samples, which was already indicated by the greater broadening of the 001 reflection in the corresponding diffraction pattern (Fig. 3 ▸).

### 3.6. Confirmation of microstructural features by complementary methods   

Thermal analysis and IR spectroscopy were used as complementary methods to confirm the microstructural parameters of the precursor sample, in particular those that are related to the intercalation of carbonate and/or water.

Taking into account the low carbon content that was determined for all samples (Table 1 ▸), it can be concluded that mainly water is intercalated between the sheets. Owing to the presence of 3R-type faults, the charge balance in the NCA-precursor material is most likely achieved by the partial substitution of hydroxide by oxide.

**Figure 11:**
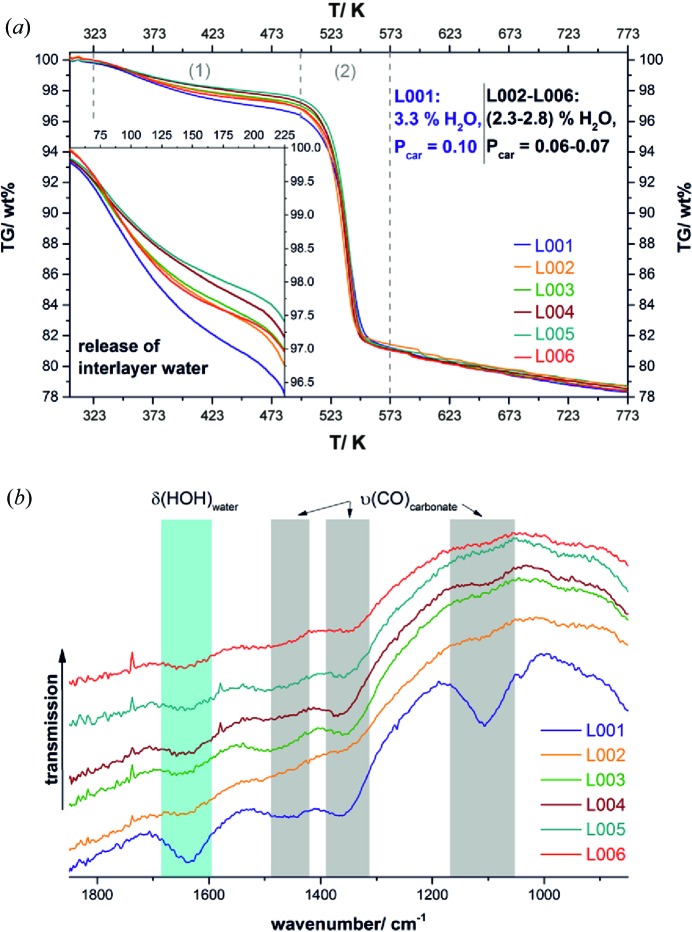
(*a*) Measured TG curves and (*b*) portions of the measured IR spectra of the NCA-precursor materials; the complete spectra are shown in Fig. S8 in the supporting information.

The TG curves of the precursor samples exhibit two mass-loss steps [Fig. 11 ▸(*a*)]. The first decomposition step starts at 323 K, partially overlaps with the second decomposition step starting at 498 K and can be assigned to the release of water. As adsorbed water was removed by an initial hold at 303 K for 30 min, the first decomposition step is associated with the release of intercalated water. During the thermal decomposition of disordered Ni(OH)_2_, a comparable thermal behaviour was observed (Ramesh, 2009 ▸). The precursor samples L002 to L006 contain almost the same amount of intercalated water, between 2.3 and 2.8 wt%, which correlates with similar numbers of intercalation faults that were determined (Table 2 ▸). Sample L001 exhibits a considerably higher mass loss during the first decomposition step (3.3 wt%), which is in accordance with the higher number of interstratification faults. The second decomposition step is associated both with the dehydration of hydroxide ions and with the decomposition of carbonate ions, as was observed during the thermal decomposition of nickel carbonate hydroxide salts (Rincke *et al.*, 2015 ▸). Therefore, the amount of intercalated carbonate cannot be extracted from the size of the second decomposition step. IR spectroscopy, however, can be used to qualitatively prove the presence of water and carbonate in the precursor materials. In Fig. 11 ▸(*b*), portions of the IR spectra of the precursor material are presented. The entire spectra are shown in the supporting information Fig. S8. At ∼1650 cm^−1^ a broad and weak band is present in all spectra [Fig. 11 ▸(*b*), cyan highlighted area], which can be assigned to the O—H bending mode of water mol­ecules on the basis of the spectral data of NiCO_3_·5.5H_2_O (Bette *et al.*, 2016 ▸), Ni(OH)_2−*x*_(CO_3_)_*x*/2_·*n*H_2_O (Minkova *et al.*, 1984 ▸) and Ni_12_(CO_3_)_8_(OH)_8_·(5–7)H_2_O (Rincke *et al.*, 2015 ▸). Bands occurring in the spectral regions of 1050–1150, 1300–1400 and 1425–1500 cm^−1^ [Fig. 11 ▸(*b*), grey highlighted areas] can be assigned to carbonate-related CO-stretching modes on the basis of the spectral data of the previously mentioned compound and on the IR spectrum of gaspeite (NiCO_3_) (Reddy & Frost, 2004 ▸). The carbonate- and water-related bands are most pronounced in the spectrum of sample L001 [Fig. 11 ▸(*b*), blue spectrum], which is an additional confirmation of the higher number of interstratification faults that were found in this sample (Table 2 ▸).

## 4. Conclusions   

Six industrially synthesized samples of the brucite-type nickel cobalt aluminium hydroxide precursor for the lithium nickel cobalt aluminium oxide, Li(Ni_1−*x*−*y*_Co_*x*_Al_*y*_)O_2_ (NCA), battery material were investigated in detail by laboratory X-ray powder diffraction. The diffraction line shapes of the materials exhibit unusual broadening and point to different kinds of disorder present in the materials. The coexistence of sharp 0*k*0 reflections and strongly broadened *hk*0 reflections indicates stacking-fault disorder, while broadening of 00*l* is caused by random intercalation of ions and molecules between the layers, *i.e.* interstratification faults. In the structures of the precursor material, shifts between the brucite-type (AγB)□(AγB)□, the CdCl_2_-type (AγB)□(CβA)□(BαC)□ and the CrOOH-type (BγA)□(AβC)□(CαB)□ stacking orders were found to occur as faults. By elemental analyses, IR spectroscopy and thermal analyses, the intercalated species could be identified mainly as water molecules and additionally a small fraction of carbonate ions. A recursion-like routine implemented in the *TOPAS* software was used to simulate diffraction patterns of the stacking-faulted NCA-precursor material as a function of the microstructure parameters, *i.e.* the probabilities for transitions between the different stacking patterns, which were fitted to the measured data. Series of simulations and subsequent refinements were performed, in which the transition probabilities were incrementally varied and the residual criterion (*R*_wp_ value) after fitting the pattern to the measured data was recorded. This procedure enabled a grid-search optimization of the three-dimensional parameter space of the microstructure. In the brucite-type stacking order of all precursor material, the same percentage of CdCl_2_- and CrOOH-like faults was found, which corresponds to identical metal concentrations (Ni:Co:Al ratios ≃ 90:5:5) of all investigated NCA precursors. For one sample, a significantly higher number of interstratification faults was found, which could be confirmed by a higher degree of broadening of the 001 reflection and a larger intercalation-water-related mass loss in the thermal analysis.

The grid-search-based optimization was found to be a robust and efficient method to fit the diffraction pattern of heavily stacking faulted materials. As industrial synthesis processes of functional materials are supposed to yield products of identical quality, which means (in terms of the NCA precursor) material with identical metal composition and identical degree of faulting, the *n*-dimensional space of the microstructure parameters that is optimized in the grid search can be further narrowed. This leads to an additional acceleration of the process. Hence, the presented routine is a promising candidate for a standardized routine for the quality control of the industrial precursor material production. As the parameter space of the grid search can be further extended, detailed microstructural investigation of materials with even more complicated faulting scenarios can now be performed. In addition, it is now possible to quantify stacking and intercalation faults in differently synthesized NCA-precursor materials and therefore to correlate the amount of faulting with the synthesis procedure in a follow-up study.

## Supplementary Material

Measured diffraction pattern of sample L002. DOI: 10.1107/S1600576719016212/po5156sup1.txt

Input file for the 3D grid search. DOI: 10.1107/S1600576719016212/po5156sup2.txt

TOPAS input file for the final refinement). DOI: 10.1107/S1600576719016212/po5156sup3.txt

Supporting information file. DOI: 10.1107/S1600576719016212/po5156sup4.pdf

## Figures and Tables

**Table 1:** Overview of the elemental analysis of the NCA-precursor material samples

Sample	Ni content (mol%)†	Co content (mol%)†	Al content (mol%)†	C content (wt%)	S content (wt%)
L001	90.3 (1)	4.8 (1)	4.9 (1)	0.11 (1)	0.03 (1)
L002	90.6 (1)	4.7 (1)	4.7 (1)	0.07 (1)	0.05 (1)
L003	90.4 (1)	4.8 (1)	4.8 (1)	0.10 (1)	0.05 (1)
L004	90.3 (1)	4.8 (1)	4.9 (1)	0.10 (1)	0.03 (1)
L005	90.2 (1)	4.7 (1)	5.1 (1)	0.09 (1)	0.03 (1)
L006	90.2 (1)	4.8 (1)	5.0 (1)	0.08 (1)	0.05 (1)

† mol% refers to the molar ratio of the cations exclusively; cations besides Ni, Co and Al were not found with quantities of more than 0.1 mol%.

**Table 2:** Refined microstructural parameters of the NCA-precursor material samples

Sample	*P_x_* (C19 fault) (%)	*P_y_* (3R fault) (%)	*P*_car_ (interstratification) (%)
L001†	0.15 (1)	0.07 (1)	0.10 (1)
L002	0.15 (1)	0.06 (1)	0.07 (1)
L003	0.15 (1)	0.06 (1)	0.07 (1)
L004	0.13 (1)	0.06 (1)	0.07 (1)
L005	0.13 (1)	0.06 (1)	0.06 (1)
L006	0.14 (1)	0.07 (1)	0.07 (1)

† For sample L001 the three-dimensional grid search in the parameter space of the transition probabilities was performed within a range of 0.10 ≤ *P_x_* ≤ 0.24, 0.00 ≤ *P_y_* ≤ 0.19, 0.07 ≤ *P*_car_ ≤ 0.12.
