# Supplementary material for: A routine for the determination of the microstructure of stacking-faulted nickel cobalt aluminium hydroxide precursors for lithium nickel cobalt aluminium oxide battery materials
Source: J Appl Crystallogr. 2020 Feb 1;53(Pt 1):76–87. doi: 10.1107/S1600576719016212 (PMC6998777; doi:10.1107/S1600576719016212)
Supplement: Supplementary file 4 [file j-53-00076-sup4.pdf]

JOURNAL OF
APPLIED
CRYSTALLOGRAPHY

Volume 53 (2020)

Supporting information for article:

A routine for the determination of the microstructure of stacking faulted nickel cobalt aluminium hydroxide precursors for lithium nickel cobalt aluminium oxide battery materials

Sebastian Bette, Bernd Hinrichsen, Daniela Pfister and Robert, E. Dinnebier

S1. Additional tables and figures

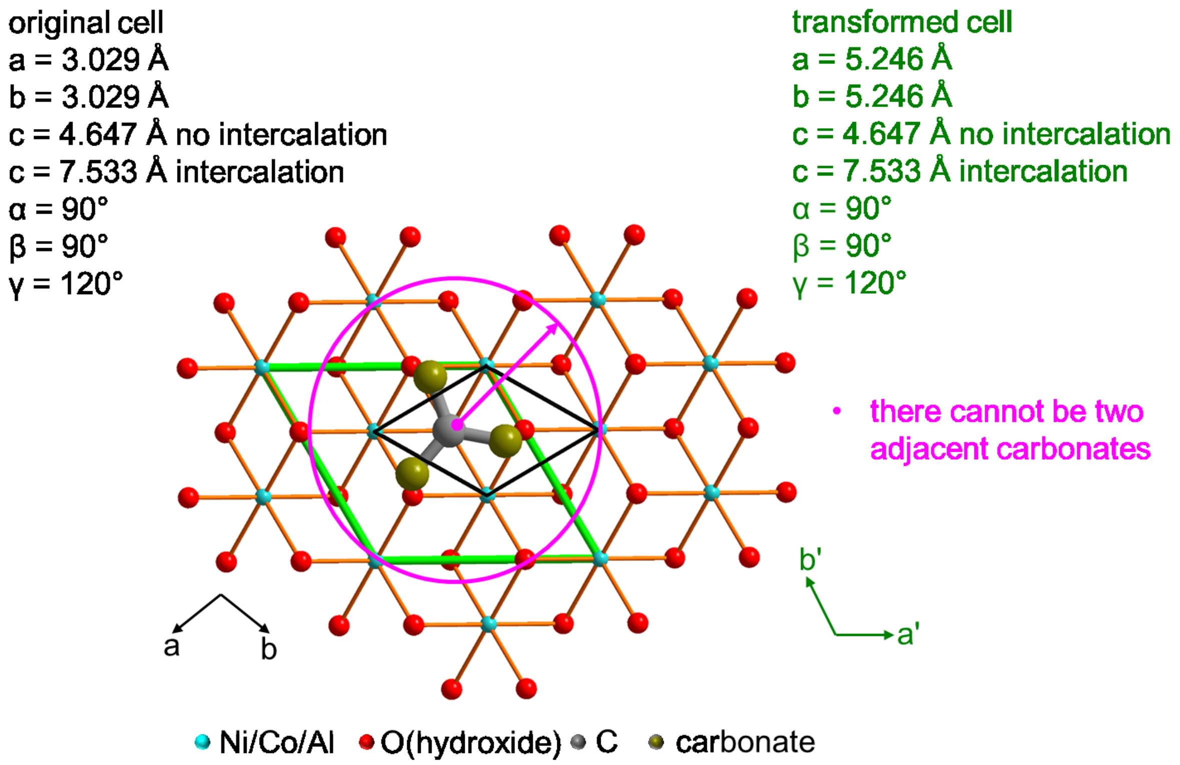

Figure S1 Illustration of the transformation of the unit cell of the brucite type NAC-precursor material in order to properly model the intercalation of carbonate ions and water molecules.

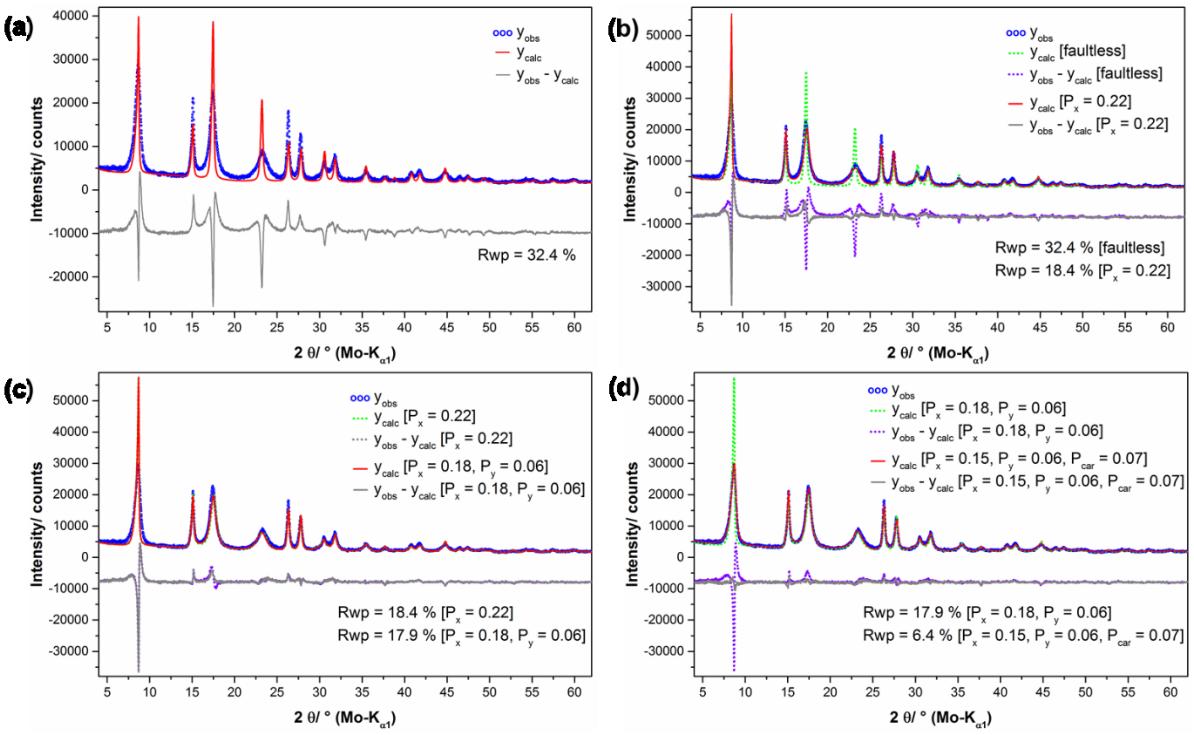

Figure S2 Comparison of the graphical results of the final Rietveld refinement of the NCA-precursor L002 by (a) using the faultless, brucite type structure, (b) at the global minimum (straight lines) of the one-dimensional grid search (Figure 7,a), the fit using the faultless structure model is presented in dashed lines, (c) at the global minimum (straight lines) of the two-dimensional grid search (Figure 7, b), the fit using the minimum of the one-dimensional parameter space is presented in dashed lines and (d) at the global minimum (straight lines) of the three-dimensional grid search (Figure 7, b), the fit using the minimum of the two-dimensional parameter space is presented in dashed lines.

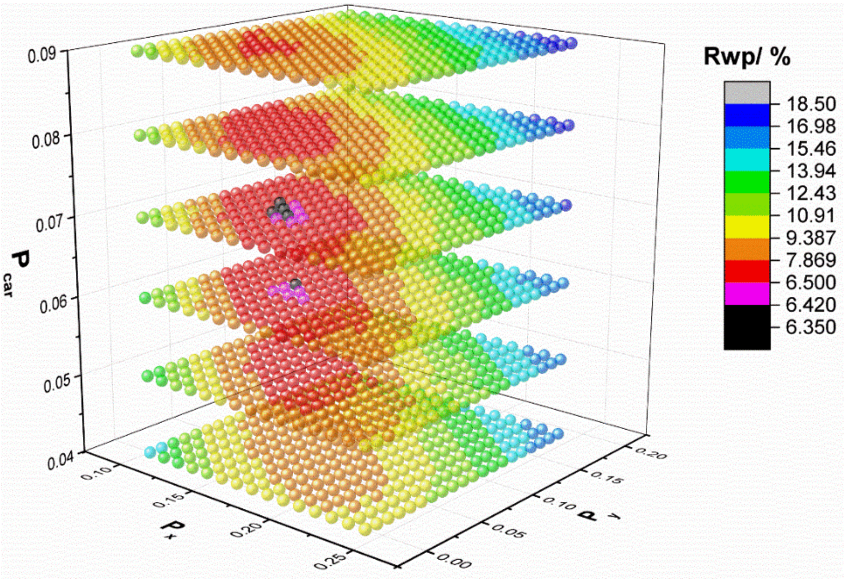

Figure S3 3-dimensional parameter space including the probabilities of C19-, 3R- and interstratification-type faults, the black highlighted points of the grid represents microstructural models that yield almost identical Rwp values and therefore may represent the global minimum of the parameter hypersurface.

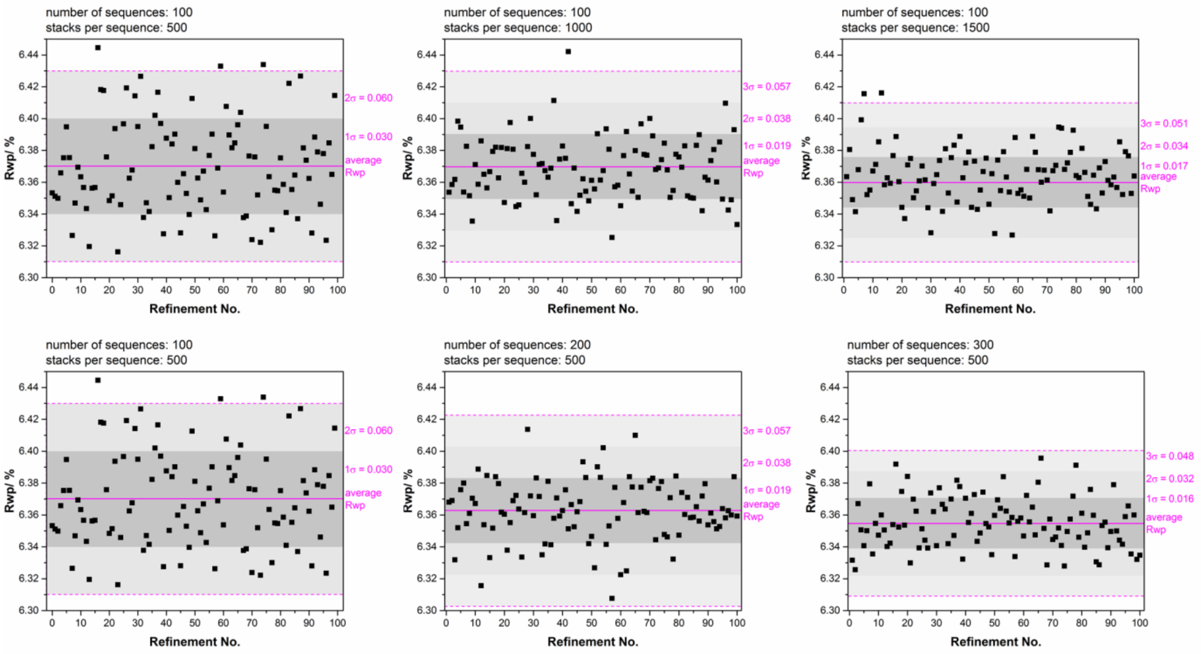

Figure S4 Evolution of the random distribution of the Rwp-factors for 100 recursively created and averaged superstructure of the NCA-precursor material while iteratively increasing the number of stacks per sequence (a-c) and while iteratively increasing the number of sequences (d-f). The ranges of the standard deviations (σ, 2 σ and 3 σ) are highlighted with backgrounds in different grey scale colours.

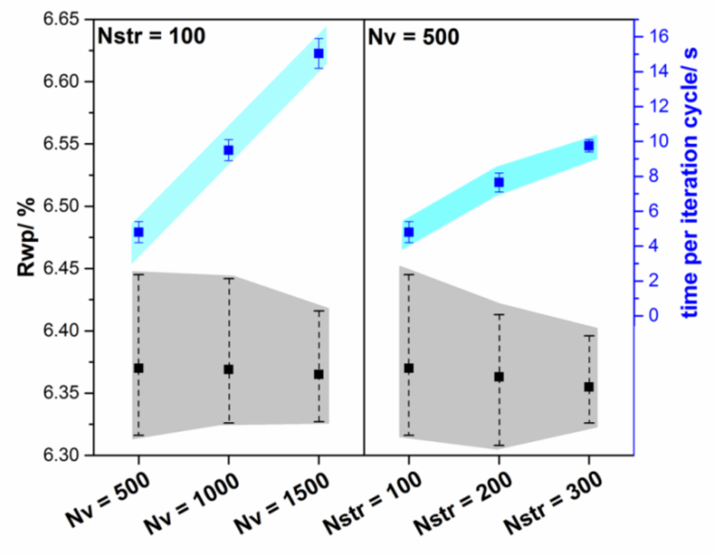

Figure S5 Evolution of the distribution of the Rwp-factors for 100 recursively created and averaged superstructure of the NCA-precursor material in dependence of the number of stacks per sequence (left) and the number of sequences (right). The average Rwp is indicated by a black square and the minimum and maximum Rwp is indicated by a horizontal black bar. The evolution of the time per iteration cycle in dependence of the number of stacks per sequence (left) and the number of sequences (right) is also presented. The average iteration time is indicated by a blue square and the variation of the iteration time is indicated by horizontal blue bars.

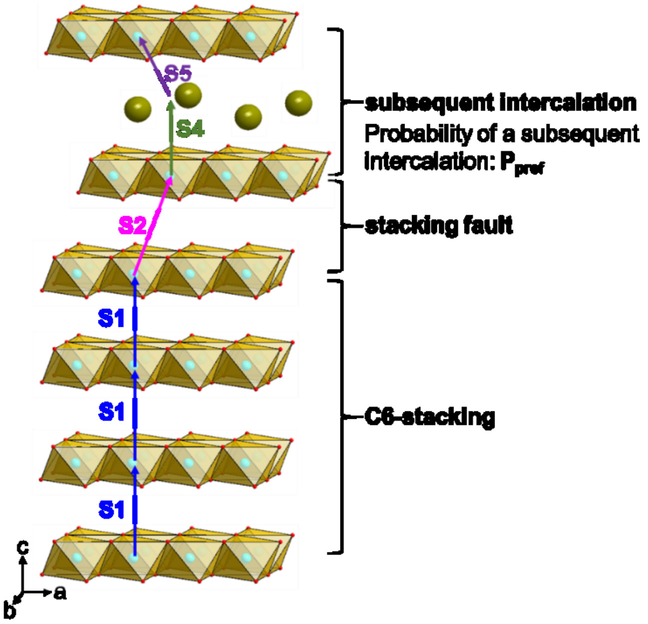

Figure S6 Illustration of a C19-type fault in the C6-type stacking that induces a subsequent intercalation.

Table S1 Applied transition probability matrices in the 3-dimensional final grid search (top) (Figure 7, c) and in the one-dimensional grid search for p_{pref} (bottom) (Figure 9, b). The stacking vectors are given in Figure 6. A, B, C, D, E, F refer to brucite-type layers, G refers to intercalation layers. Layers D, E and F had to be added as the c-component of the stacking vector cannot be varied within a row of the transition probability matrix in the TOPAS syntax.

from↓/to→	A	B	C	D	E	F	G
**A**	$1-p_x-p_y-p_{car}$, **S1**	p_x, **S2**	p_y, **S3**	$p_{car}\cdot(1-p_x-p_y)$, **S1**	$p_{car}\cdot p_x$, **S2**	$p_{car}\cdot p_y$, **S3**	0
**B**	$1-p_x-p_y-p_{car}$, **S1**	p_x, **S2**	p_y, **S3**	$p_{car}\cdot(1-p_x-p_y)$, **S1**	$p_{car}\cdot p_x$, **S2**	$p_{car}\cdot p_y$, **S3**	0
**C**	$1-p_x-p_y-p_{car}$, **S1**	p_x, **S2**	p_y, **S3**	$p_{car}\cdot(1-p_x-p_y)$, **S1**	$p_{car}\cdot p_x$, **S2**	$p_{car}\cdot p_y$, **S3**	0
**D**	0	0	0	0	0	0	1, S4
**E**	0	0	0	0	0	0	1, S4
**F**	0	0	0	0	0	0	1, S4
**G**	0	0	$(1-p_{car})$, **S5**	0	0	p_{car}, **S5**	0

from↓/to→	A	B	C	D	E	F	G
**A**	$1-p_x-p_y-p_{car}$, **S1**	$p_x-p_x\cdot(1-p_{car})\cdot p_{pref}$, **S2**	$p_y-p_y\cdot(1-p_{car})\cdot p_{pref}$, **S3**	$p_{car}\cdot(1-p_x-p_y)$, **S1**	$p_{car}\cdot p_x+p_x\cdot(1-p_{car})\cdot p_{pref\ x}$, **S2**	$p_{car}\cdot p_y+p_x\cdot(1-p_{car})\cdot p_{pref}$, **S3**	0
**B**	$1-p_x-p_y-p_{car}$, **S1**	p_x, **S2**	p_y, **S3**	$p_{car}\cdot(1-p_x-p_y)$, **S1**	$p_{car}\cdot p_x$, **S2**	$p_{car}\cdot p_y$, **S3**	0
**C**	$1-p_x-p_y-p_{car}$, **S1**	p_x, **S2**	p_y, **S3**	$p_{car}\cdot(1-p_x-p_y)$, **S1**	$p_{car}\cdot p_x$, **S2**	$p_{car}\cdot p_y$, **S3**	0
**D**	0	0	0	0	0	0	1, S4
**E**	0	0	0	0	0	0	1, S4
**F**	0	0	0	0	0	0	1, S4
**G**	0	0	$(1-p_{car})$, **S5**	0	0	p_{car}, **S5**	0

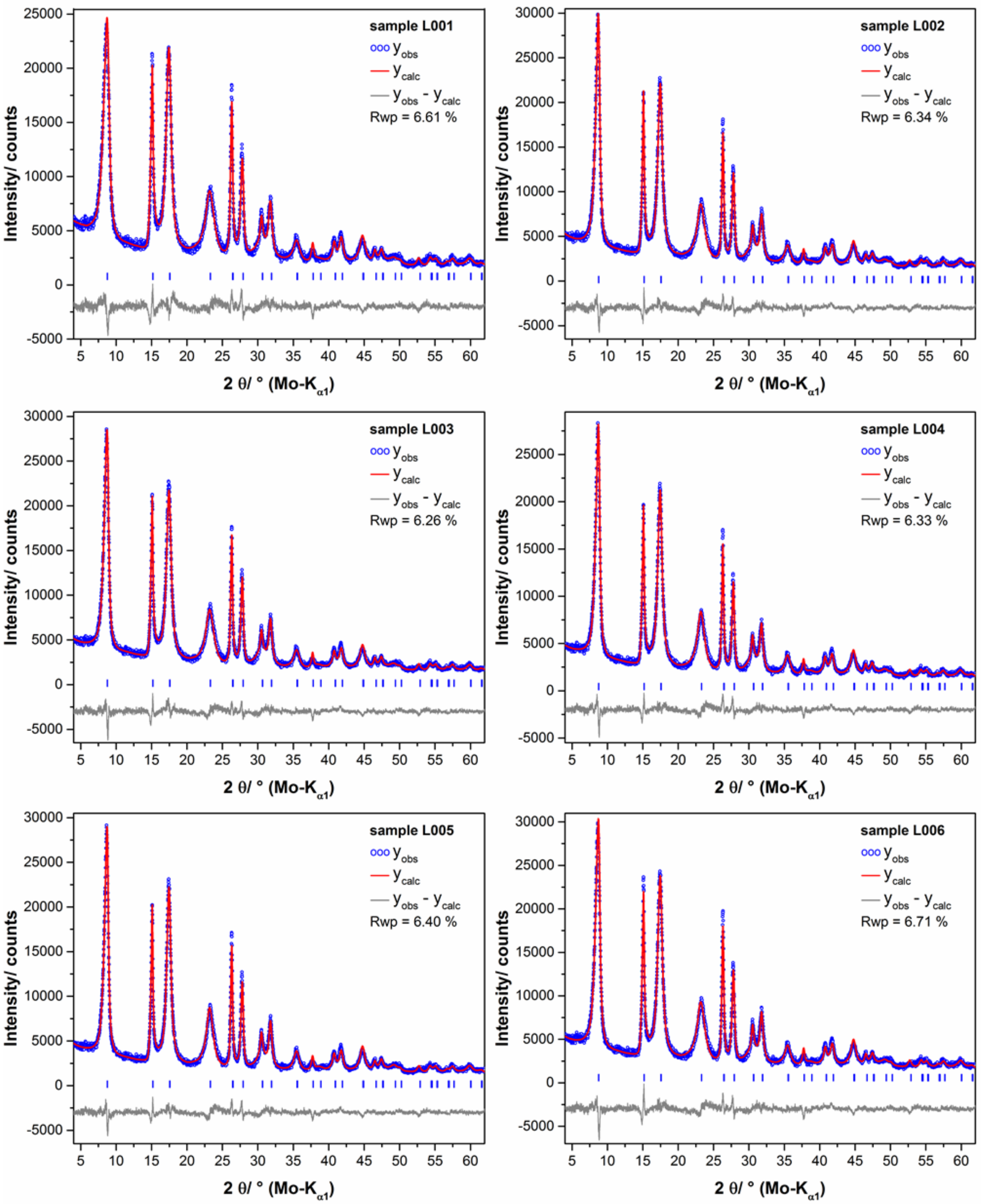

Figure S7 Graphical results of the final Rietveld refinements of the NCA-precursor materials by averaging 100 supercells with 500 layers each and using transition probabilities given in **Error! Reference source not found.**. The reflection positions of the ideal faultless brucite-type structure are given in each graph.

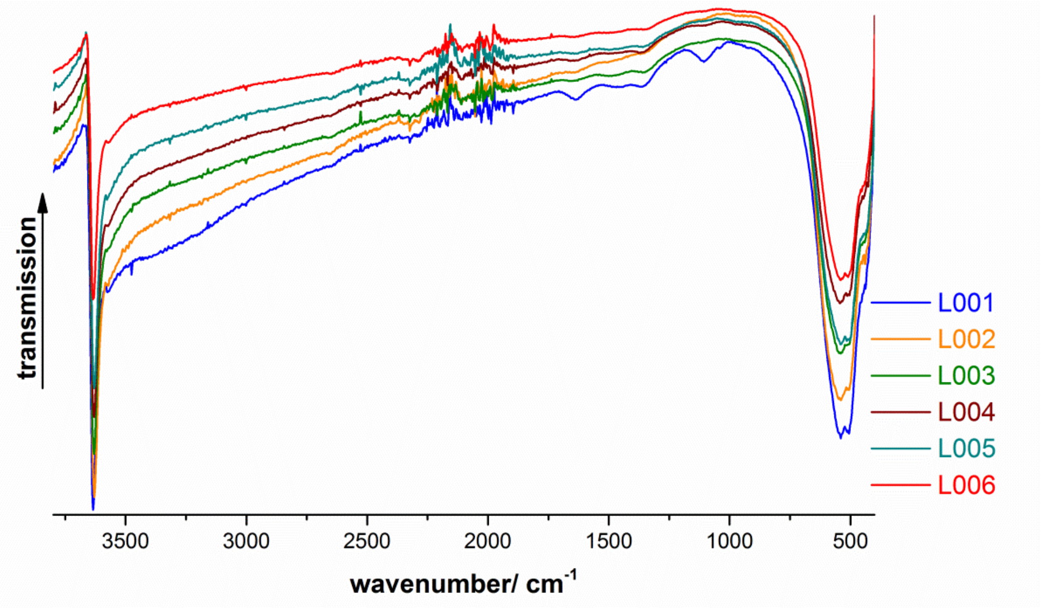

Figure S8 IR-spectra of the investigated NCA-precursor materials

S2. Demonstration of the grid search in TOPAS syntax

S2.1. One-dimensional grid-search

In order to perform a grid-search optimization in any parameter space, a TOPAS input-file has to be executed many times. This can be realized by the "num-runs" command (Table S2). The parameters, which should be optimized, are varied from one run to another by an incremental value. By including "##Run_Number##" into the syntax the run number is used as running index that increases from one run to the next one, as it is suggested for performing a series of simulations (Dinnebier *et al.*, 2019). For a one dimensional grid search, the input file was executed 101 times, starting with run number 0 and ending with run number 100. The actual run number divided by 100 was used as parameter value for P_x. Hence, in the one dimensional grid search, P_x was varied from 0.00 to 1.00 in 0.01 increments (Table S2). With a conventional PC or notebook, the one-dimensional grid search of the NCA-precursor samples takes approx. 40-50 minutes.

Table S2 Selected commands in TOPAS syntax that were used to perform a one-dimensional grid search in the parameter space of the transition probabilities.

TOPAS-syntax	Explanation
num_runs 101	The input file is executed 101 times, i.e .101 grid points are investigated.
seed	Before execution of the input file the random number generator creates a new set of random numbers
prm !px =(##Run_Number##)/100;	The run number is used as a running index, starting at 0, from one run to the next one the parameter P_x is increased by 0.01, which lead to $P_x = 1$ in the last run.
out "grid-1D.txt" append<br>Out(Get(r_wp),\t%11.5f)<br>Out(px, "\t%11.5f\n")	The Rwp value and the parameter value of P_x are stored in a separate ASCII file, as the INP-file is not modified and not OUT file is created when "num_runs" is used

S2.2. Two-dimensional grid-search

In a two dimensional grid search, two parameters: P_x and P_y are optimized simultaneously, i.e. a grid has to be created that contains all points from $P_x = 0.01$ to $P_x = 1.00$ and $P_y = 0.00$ to $P_y = 1.00$. If the parameters are varied in 0.01 increments, this grid will contain 10099 points (excluding $P_x = 1.00$, $P_y = 0$). In order to search all points, this grid is subdivided into 101 rows with each row containing 100 points (Table S3). Within a row P_x is varied from 1.00 to 0.00 in 0.01 increments and P_y is kept constant. When the grid search is completed in one row, i.e. $P_x = 0.00$, another row is started, which means that P_y is increased by 0.01 and P_x switches back to 0.99. By using the "Round" command the grid is divided into 101 rows ("rownumber", Table S3) consisting of 100 points. In addition, the "Round" command provides integer value for the "rownumber" running index that is increased by 1 after 100 runs. With a conventional PC or notebook, the two-dimensional grid search of the NCA-precursor samples took between (7 and 8) hours.

Table S3 Selected commands in TOPAS syntax that were used to perform a two-dimensional grid search in the parameter space of the transition probabilities.

TOPAS-syntax	Explanation
num_runs 10098	The input file is executed 10099 times; i.e .10099 grid points are probed, starting at num_runs = 0.
prm rownumber = Round((##Run_Number##-49)/100);	The running index parameter "rownumber" is created that is in dependence on the Run_Number index. The round command provides an integer value for this parameter. The rownumber is increased by 1 after 100 runs. This provides a subdivision of the 10099 runs into 101 sets with 100 runs each.
prm !px = ((rownumber*100+100)-(##Run_Number##+1))/100;	The transition probability P_x is varied from 1.00 to 0.01 in 0.01 increments during a set of 100 runs. At the beginning of a new set of 100 runs, P_x is set back to 1.00.
prm !py = rownumber/100;	The transition probability P_y is varied from 0.00 to 1.00 in 0.01 increments. Within a set of 100 runs, P_y is kept constant.
out "grid-2D.txt" append<br>Out(Get(r_wp),\t%11.5f")<br>Out(px, "\t%11.5f")<br>Out(py, "\t%11.5f\n")	The Rwp value and the parameter values of P_x and P_y are stored in a separate ASCII file

S2.3. Three-dimensional grid-search

Extending a two-dimensional grid to a three-dimensional one in order to optimize both P_x, P_y and P_{car} requires both a further subdivision of the running index and an increase of the total number of runs. As a full three dimensional grid search with all parameters varying from 0.00 to 0.99 in 0.01 increments contains 100x100x100 = 10000000 points and will take several weeks on a conventional PC or notebook, the grid search was limited to an excerpt of $0.10 \leq P_x \leq 0.24$, $0.00 \leq P_y \leq 0.19$, $0.04 \leq P_{car} \leq 0.09$ in which the global minimum was expected based on the two dimensional grid search and which only contains 1800 points. This grid search can be realized by combing several two-dimensional grid searches in the P_x-P_y parameter space (Table S3). Hence the three dimensional grid is divided into two subdivisions:

1. rows that contain 20 points in which P_y is decreased from 0.19 to 0.00 in 0.01 increments and P_x and P_{car} are kept constant;

2. levels that consist of 15 rows, within a level P_x is increased from row to row from 0.10 to 0.24 in 0.01 increments and P_{car} is kept constant,

3. the entire parameter space is divided into six levels, P_{car} is increased from level to level from 0.04 to 0.09 in 0.01 increments.

The TOPAS syntax for the three dimensional grid search is presented in Table S4. With a conventional PC or notebook, the two-dimensional grid search of the NCA-precursor samples took between (2 and 3) hours.

Table S4 Selected commands in TOPAS syntax that were used to perform a three-dimensional grid search in the parameter space of the transition probabilities within a range of $0.10 \leq P_x \leq 0.24$, $0.00 \leq P_y \leq 0.19$, $0.04 \leq P_{car} \leq 0.09$.

TOPAS-syntax	Explanation
num_runs 1799	The input file is executed 1800 times; i.e .1800 grid points are probed, starting at num_runs = 0.
prm row = Round((##Run_Number##*5-49)/ 100);	The running index parameter "row" is created that is always increased by 1.0 after the input file was executed for 20 times. This provides a subdivision of the 1800 runs into 91 sets with 20 runs each.
prm levels = Round((##Run_Number##/300)-0.49999);	For further subdivision of the running indices, an additional running index is introduced. The running index parameter "levels" is always increased by 1.0 after the input file was executed for 300 times. This provides a subdivision of the 1800 runs into 6 sets with 300 runs each.
prm rownumber = row-(levels*15);	The running indices "row" providing a subdivision of sets of 20 runs and "levels" providing a subdivision of sets of 300 runs are linked by the parameter "rownumber"
prm !px = rownumber/100+0.1;	The transition probability P_x is varied from 0.10 to 0.24 in 0.01 increments. Within a set of 20 runs, P_x is kept constant.
prm !py = (##Run_Number##/100)-(row*(20/100));	The transition probability P_y is varied from 0.00 to 0.19 in 0.01 increments during a set of 20 runs. At the beginning of a new set of 20 runs, P_y is set back to 0.00.
prm !pcar = 0.04 + levels/100;	The transition probability P_{car} is varied from 0.04 to 0.09 in 0.01 increments. Within a set of 300 runs, P_{car} is kept constant.
out "grid-2D.txt" append<br>Out(Get(r_wp),\t%11.5f")<br>Out(px, "\t%11.5f")<br>Out(py, "\t%11.5f")<br>Out(pcar, "\t%11.5f\n")	The Rwp value and the parameter values of P_x, P_y and P_{car} are stored in a separate ASCII file
